# Knowing, Being and Co-Constructing an Age-Friendly Tāmaki Makaurau Auckland

**DOI:** 10.3390/ijerph17239136

**Published:** 2020-12-07

**Authors:** Judy Blakey, Janet Clews

**Affiliations:** 1 Comprehensive Care PHO, Auckland 0632, New Zealand; 2 The Trusts Community Foundation Ltd., Auckland 0650, New Zealand; jernie@xtra.co.nz

**Keywords:** age-friendly, Tāmaki Makaurau Auckland, Polynesian population, relational leadership, Te Tiriti o Waitangi

## Abstract

A third of Aotearoa New Zealand’s increasingly ageing population resides in Tāmaki Makaurau Auckland. This most populous cosmopolitan urban area in the country is also home to the largest Polynesian population of any global city. Sprawling across a North Island isthmus inclusive of Hauraki Gulf islands, 70% of the city region is rural, whilst almost 90% of the ethnically diverse residents live in urban areas. Members of Auckland Council’s Seniors Advisory Panel (SAP) advocated for, and in 2018 secured unanimous support from the governing body to resource an Age-friendly City (AFC) Project. This case study inquiry applied bricolage methodology to provide diverse contextual perspectives of this unique Polynesian setting, prior to exploring interview narratives of three SAP members who served two consecutive terms (six years) as AFC advocates. Weaving insights gleaned from their interview transcripts responding to relational leadership prompts about their age-friendly advocacy with the findings from the council’s AFC Community Engagement report highlighted the achievements and challenges of the evolving AFC Project. Service-learning recommendations include co-developing: (1) A sustainable co-governance framework for an independent steering group that embodies the values and principles of Te Tiriti o Waitangi to enable empowered active ageing for all residents; (2) A succession plan that enables the timely transfer of knowledge and skills to empower incoming SAP members.

## 1. Introduction

An increasing number of countries are responding to the challenges associated with demographic changes and population ageing; challenges that have been amplified by the disruptive impact of the coronavirus pandemic [1]. Planning for the diverse implications of these changes in longevity and age structure has highlighted the need for greater investment in improving how societies age [2].

Combining demographic, economic, health and social gerontology models that explore optimizing participation in various roles and domains of life [3], the multi-dimensional concept of Active Ageing [4] has provided the most useful contemporary policy response to demographic ageing [5]. Emphasizing the link between activity and health that challenges the deficit model [6], expectations related to the three original Active Ageing pillars of participation, health and security identified by the World Health Organization (WHO) [7] are typically framed by researchers and policy makers, rather than older people themselves [8]. An emergent “good active ageing” conceptual and policy framework has added learning, ethical and moral foundations prescient within the context of deep technological, economic and social change, to create an additional Active Ageing pillar [9].

To translate the Active Ageing policy agenda into urban environments [10] the WHO initially collaborated with partners in 35 cities from high, middle, and low-income countries [11]. Guided by the Vancouver protocol’s methodological requirements, the project identified eight core age-friendly features that supported the dynamic ecological interplay between individual adaptation and environmental alteration [12] to optimize residents functioning as they aged. The resulting WHO Global Age-friendly Cities Guide [13] and companion checklist [14] captured the imagination of planners and connected with the zeitgeist of ageing communities across the globe. Currently the WHO’s Global Network comprises 1000 cities and communities in 41 countries that impacts the lives of 240 million people [15].

The growth in the WHO’s Global Network of Age-friendly Cities and Communities (AFCC) [16] has in turn generated a vast number of research publications [17,18,19,20]. Torku et al.’s (2020) systematic review of 98 AFCC publications observed that although AFCC are driven by community-led processes, major initiatives demand strong and committed political leadership and ”top-down” support for implementing the ‘bottom-up’ community development age-friendly action plans [20]. A review commissioned to support the implementation of AFCC in Aotearoa New Zealand concluded that AFCC processes and initiatives need to have in-built flexibility that recognize local geographic and demographic diversity, and include the needs of indigenous Māori, Pacific and other cultural groups [19]. The review also stated that older people should be involved in all stages of the development of processes and plans for AFCC, and that their voices should be heard with respect and dignity [19].

In the UK, a Manchester-based age-friendly movement manifesto articulated a new urban agenda that focused on issues concerned with inequality and empowerment [10]. This noted the need for creative participatory research methods [21] and the development of interdisciplinary networks to generate comparative insights [22] and critical awareness of those who may be socially marginalized in the face of urban change.

The global pandemic has highlighted the need to protect older people’s health and wellbeing. However, in Aotearoa New Zealand the downstream impact of past and contemporary colonization has caused and sustained intergenerational social, economic and health equity disadvantages, that have resulted in higher hospitalization and death rates for Māori during previous pandemics [23]. Prioritizing mitigating COVID-19′s impact without increasing existing health inequities, the current pandemic response has nudged health and wellbeing service providers to examine if continuation under a business-as-usual model will preferentially benefit Pākehā (European) New Zealanders, and fail to protect Māori from the worst outcomes [23]? That question informs this research, which embraces community-based participatory research methodology [24] to co-construct narratives of Tāmaki Makaurau Auckland’s evolving Age-friendly City (AFC) Project from the perspectives of four older community leaders and AFC champions. The narratives offer contextual details of the city’s sprawling location and demographic contours of her older residents; summarise achieving AFC Project milestones that challenged ageism and created visibility of the needs of older residents in Auckland Council’s research, planning and policy work; and articulate the community leaders’ perceptions of the challenges and risks (both personal and organizational) associated with the AFC Project.

## 2. Materials and Methods

Bricolage research methodology embraces the complexity of researching lived experiences by applying critical methods that address the plurality and power dynamics of knowledge production [25,26]. In this research context the authors-as-research-participants explore interpretive bricolage as an activist scholarship inquiry process [27] to develop and inform a fuller understanding of multiple perspectives and texts that reflect diverse voices and information sources pertinent to Tāmaki Makaurau Auckland’s emergent Age-friendly Action Plan [19,28,29].

Six contiguous sections reveal the selected contextual and methodological layers within this bricolage inquiry [30]. The first section introduces Tāmaki Makaurau Auckland’s unique Polynesian setting [31], including the city’s governance framework [32], Aotearoa New Zealand’s founding indigenous document, *Te Tiriti o Waitangi* [27,33] and salient demographic contours of the city’s residents [19]. The second section provides historical insights about the evolving Age-friendly Auckland Project [34,35] highlighting the two SAP’s achievements. Section three situates and elaborates on methodological challenges associated with autoethnographic inquiries [36] as a segue to the fourth section describing the processes involved in using relational leadership interview prompts [37,38] to elicit the narratives of older co-researchers active in progressing Tāmaki Makaurau Auckland’s evolving Age-friendly City (AFC) agenda [39]. Section five describes the research participants, and section six explains the methods used to analyse the interview transcripts.

### 2.1. The Place: Our Unique Polynesian Setting

Māori, who are the indigenous people (mana whenua) of Aotearoa New Zealand, have lived in Tāmaki Makaurau for over 1000 years [40]. Tāmaki is the Māori name for Auckland and means desired by many; a name that alludes to the desirable qualities of the land, volcanic cones, mountains, waters, harbours and 3700 km coastline that first attracted Māori to settle in the isthmus. Founded in 1840 as a colonial settlement by British naval officer William Hobson, the clusters of modest dwellings and trading ports have evolved over the intervening 180 years into one of the largest urban areas in Australasia [31].

Two harbours, an extensive volcanic field and two mountain ranges have shaped Auckland’s urban morphology, which has been characterised by sprawling low-density development, with residents reliant on private motor vehicles for mobility [41]. The new millennium has ushered in more medium density housing developments and apartments, particularly in the city centre, but also in fringe areas. Transport infrastructure has also received considerable investment, bringing train into the city and a busway to Auckland’s North Shore, with a significant increase in public transport patronage [31].

#### 2.1.1. Auckland Council Governance Processes

In 2010 central government’s Royal Commission on Auckland Governance recommended the amalgamation of eight existing territorial authorities into a single Auckland Council [42]. The resulting “Super City” shares governance functions across a governing body (comprising an elected mayor and 20 councillors) that focuses on regional strategic decisions and 21 local boards whose members focus on local issues, activities and facilities. In addition, a number of council controlled organisations deliver a range of services to residents and visitors (e.g., transport, water, property development and management, regional facilities, and tourism and events). An Independent Māori Statutory Board ensures the views of local Māori residents—inclusive of both mana whenua (residents who have ancestral relationships with at least one of the 19 designated tribal groupings within Tāmaki Makaurau) and matāwaka (residents whose tribal ancestors settled outside the region) —are taken into account [32].

Auckland is experiencing sustained population growth, and future projections indicate the 2018 Census measure of 1.6 million residents will exceed 2 million by 2030, maintaining the demand for equitable and accessible housing, transport and employment solutions [31]. Ethnically and culturally diverse, the city is currently home to people from over 120 ethnicities [43]. The Auckland Plan 2050 highlights the rapid growth in the numbers and proportion of older residents (included because of the strong advocacy of the Seniors Advisory Panel’s March 2018 “Focus on the Future” forum, amongst other consultation feedback), which will impact on the demand for and provision of health, support, and transport services [44]. Auckland Council provides opportunities to engage diverse community perspectives on a range of regional policies, plans and strategies through meetings and workshops with demographic and sector advisory panels [45]. The panel members also advise the council about any matters of particular concern relevant to their respective communities [32].

The Auckland Plan 2050′s shared values of Atawhai (Kindness, generosity), Kotahi (strength in diversity), Auaha (creativity, innovation), Pono (integrity) and Taonga tuku iho (future generations) [46] (p. 23) express the contemporary ethos of Auckland’s unique cultural heritage. In addition, the explicit focus within the plan on developing and nurturing partnerships with local Māori to position Māori aspirations at the heart of ethical strategic actions breathes life into Auckland Council’s statutory obligations to honour the principles articulated in Aotearoa New Zealand’s founding document, *Te Tiriti o Waitangi (te Tiriti)/The Treaty of Waitangi (The Treaty)* [46] (p. 58).

#### 2.1.2. Te Tiriti o Waitangi

Signed in 1840 by Māori leaders and representatives of the British Crown, the Auckland Plan 2050 acknowledges the specific obligations that Auckland Council has as a delegate of the Crown to Māori under *te Tiriti* [46] (2050 Print). Māori political action has ensured that *te Tiriti* has assumed a progressively significant place in legislation, jurisprudence, social and economic life in Aotearoa New Zealand [47] (Barnes). Treaty principles that have emerged have been expressed through a range of courts and the Waitangi Tribunal [46], however, integrating them into effective policies and strategies that identify and address the underlying social determinants of systemic inequalities resulting from colonization continues to challenge sector agencies [48,49].

The preamble to *te Tiriti* foreshadows the content of the articles that follow by focusing on authentic relationship building processes (whanaungatanga) which recognize each party’s sphere of influence and ways of relating to facilitate power sharing, mutual respect and understanding [27]. Recognising the crucial links between *te Tiriti* and effective health promotion practice, Berghan et al. (2017) state: “*Te Tiriti o Waitangi (te Tiriti)* legitimises settler presence in Aotearoa New Zealand and governance by the British Crown. Therefore, *te Tiriti* must lie at the heart of ethical health promotion in this country.” [27] (p. 8). They explain that health promotion practices enabling people to take control over their health align with strategic principles and values expressed in *te Tiriti,* particularly developing reciprocal partnerships, providing active protection, empowering informed decision making, enabling self-determination and evaluating equitable outcomes.

Building relationships through sharing experiences and working collaboratively provides a strong basis for an intercultural Auckland [46] (p. 32). In 2018, the neighbouring city of Hamilton, which is 130 km south of Auckland, became the first New Zealand and 600th global city to join the WHO’s AFCC Global Network [16,50]. Similar social processes are implicit in the community development activities that preceded, informed and shaped the city of Hamilton’s age-friendly action plan [51].

#### 2.1.3. The People: Demographic Contours

The Auckland Plan 2050 reports that Tāmaki Makaurau Auckland has always been ethnically diverse, and that migration patterns in the past two decades have substantially increased the numbers of Asian People living in Tāmaki Makaurau Auckland [52]. Data presented in Table 1 reveal the comparative percentages of New Zealand and Tāmaki Makaurau Auckland residents who self-identified with one of the four main ethnic groups (European, Asian, Māori and Pacific Peoples) in the 2018 Census. The term “Asian people” is a very broad category that includes a range of national origins and ethnic identities with Chinese, Indian, Korean, Filipino and Sri Lankan communities comprising the five largest Asian groups in Tāmaki Makaurau Auckland [52]. The Middle Eastern/Latin American/African (2.3%) and ”Other ethnicity” (1.1%) groups in Tāmaki Makaurau Auckland are not included in Table 1 due to the relatively small percentages of people included in these extremely diverse ethnic categories [53]. The adjacent Aucklanders’ median ages column reveals that both the Māori (24.9 years) and Pacific Peoples’ (24.0 years) are relatively youthful populations in comparison with the European (oldest at 39.4 years) and Asian (31.9 years) populations.

Table 2’s presentation of median ages across Tāmaki Makaurau Auckland’s 21 local boards alongside the percentage and numbers of residents aged 65 plus provides a more nuanced appreciation of the geographic dispersion of ethnic ageing across the region. The data reveal that larger numbers of older European and Asian residents reside on Auckland’s Hauraki Gulf Islands and suburbs north and east of the central city [52]; while the more youthful Māori and Pasifika residents have gravitated to suburbs in the south and west. 2018 Census data show nearly a quarter (23.4%) of the total Māori population in Aotearoa New Zealand live in Tāmaki Makaurau, with one in nine (11.5%) city residents identifying as Māori. Only five percent of Māori were aged 65 years or older; however, the 45.9% growth in that age group was considerably faster than the 26.9% growth rate for the overall usually resident Māori population [54].

Māori migration from rural to urban areas accelerated at the end of World War II, encouraged by government policies and incentives to stimulate post war industry and boost employment [55]. The downstream impact of these policies has meant that many urban Māori have had to face significant disadvantages such as inadequate housing, racial discrimination, unemployment and the erosion of their language, culture and identity when they were discouraged from speaking their language (Te Reo Māori) in schools or workplaces [56].

Barnes and McCreanor’s (2019) essay addresses the stories of the historical trauma Māori experienced as evidenced in the trajectory of population health disparities over time, which reveal how migrant settler communities flourished at the expense of local Māori. They write: “The lived experience of injustice, brutality, deprivation, and marginalisation has been transmitted across multiple generations, aggravated by land loss, economic disempowerment, poverty, disease and racism that are reflected in diverse statistics of disparity and particularly as we have agreed, in health and wellbeing.” [47] (p. 23). The accumulative impact of these traumatic multi-generational losses is evident in the lower life expectancy statistics for both Māori and Pacific Peoples compared with European and Asian groups in Aotearoa New Zealand (See Table 1).

**Table 1:** Comparative ethnic profiles in New Zealand and Tāmaki Makaurau Auckland showing ethnic groups’ median ages for Auckland residents and population level life expectancy at birth.

Ethnic Groups	2018 Census % of New Zealanders Who Self-Identified ^a^	2018 Census % of Aucklanders Who Self-Identified ^b^	2018 Census Aucklanders’ Median Ages (Years) ^c^	2012–2014 NZ Female Life Exp. at Birth (Years) ^d^	2012–2014 NZ Male Life Exp. at Birth (Years) ^d^
European	70.2%	53.5%	39.4	83.9	80.3
Asian	15.1%	28.2%	31.9	87.2	84.4
Māori	16.5%	11.5%	24.9	77.1	73.0
Pacific Peoples	8.1%	15.5%	24.0	78.7	74.5

Data sources include: ^a^ Stats NZ 2018 Census population and dwelling counts [57]. ^b^ Stats NZ 2018 Census Place Summaries Auckland Region [53]. ^c^ Auckland Council Research and Evaluation Unit (RIMU): *Māori in Tāmaki Makaurau* [54]. ^d^ Ministry of Social Development: *Health Life expectancy at birth report* with 2012–2014 data [58].

Over the past 150 years Pacific Peoples have also settled in Tāmaki Makaurau Auckland. During that period of time two significant waves of migration in the 1960s and later the 1970s saw the initial small immigrant community in search of employment, educational opportunities and/or for family reasons, grow into one of considerable size and social significance [59]. Mainly from Polynesian islands with historical connections to Aotearoa New Zealand (such as Samoa, Tonga, the Cook Islands and Niue), the Pacific population is the youngest of all the main ethnic groups and continues to grow mainly through natural increase, but also migration. Depending on the future impact of climate change in the Pacific nations current migration projections could change. The 2018 Census data show that two thirds (63.9%) of Pacific Peoples live in Auckland, compared with a quarter (25.5%) of those who identified as European [60]. The nearly one quarter of a million Auckland residents who identified as Pasifika Peoples represented a 25.1% increase since 2013. As with Māori, a relatively small proportion of the Pasifika population (5.6%) is 65 years or older, but the 38.7% increase in older Pacific Peoples is much faster than the overall growth rate for the entire Auckland-based Pasifika population [60].

Salesa has observed that Auckland is often called the world’s largest Polynesian city, but that in reality most residents lived next door to that city [61]. Table 2 data provide glimpses of why Salesa stated that Tāmaki Makaurau Auckland was heading towards a population of “old white people and young brown people: the fastest growing group of babies are Māori and Pasifika, and the caregivers for elderly Pākehā (Europeans) will be Pasifika, Māori and Asian” [61] (p. 2). Salesa concluded that policy implications for our shared Pacific future would require greater attention also be paid to young Māori and Pasifika People’s education, training, health and wellbeing [62].

**Table 2:** Tāmaki Makaurau Auckland’s 21 local boards ranked from oldest to youngest by median age, alongside the percentage and the actual numbers of residents aged 65+ years ^1^.

#	Local Board (Island/Location) (North/West/South/East/Central)		Median Ages (Years)	% of Residents Aged 65+	Residents Aged 65+
1	Aotea/Great Barrier	(Island)	52.9	24.5%	234
2	Waiheke	(Island)	46.5	20.9%	1893
3	Rodney	(North)	42.2	16.7%	11,088
4	Hibiscus and Bays	(North)	41.2	17.6%	18,357
5	Franklin	(South)	40.5	15.1%	11,304
6	Orakei	(East)	40.0	15.8%	13,329
7	Devonport-Takapuna	(North)	39.4	16.3%	9426
8	Howick	(East)	37.3	13.5%	19,086
9	Waitakere Ranges	(West)	36.8	10.4%	5403
10	Upper Harbour	(North)	35.7	12.1%	7605
11	Kaipatiki	(North)	34.8	11.6%	10,257
12	Whau	(West)	34.4	12.2%	9618
13	Albert-Eden	(Central)	34.2	10.1%	9945
14	Puketapapa	(West)	33.8	12.1%	7014
15	Henderson-Massey	(West)	33.1	10.4%	12,336
16	Maungakiekie-Tāmaki	(East)	33.0	10.6%	8115
17	Papakura	(South)	32.0	10.5%	6069
18	Waitematā	(Central)	31.4	7.9%	6546
19	Manurewa	(South)	29.5	8.3%	7980
20	Otara-Papatoetoe	(South)	29.1	8.2%	6963
21	Māngere-Otahuhu	(South)	29.0	8.5%	6642
	Tāmaki Makaurau Auckland		34.7	12.0%	189,210

^1^ Auckland Council Research and Evaluation Unit (RIMU). 2018 Census Results, local board and special area information sheets [63].

### 2.2. Ka Mua, Ka Muri (A Māori Proverb That Means “Walking Backwards into the Future”): Co-Constructing Our Age-Friendly Futures

In 2014 Auckland Council convened the first Seniors Advisory Panel (SAP) (2014–2016). In their final report the SAP noted, “from the outset, the panel made it clear that Auckland should become part of the World Health Organisation (WHO) international network of Age-friendly Cities, if it was to be the world’s most liveable city.” [34] (p. 3). Despite encountering political and administrative roadblocks that thwarted them during their term the SAP advocated for the rapidly increasing number of seniors who were contributing to Auckland’s expanding diversity. Their advocacy resulted in Auckland Council commissioning research to gauge seniors’ wellbeing and the production of *Older Aucklanders: A Quality of Life Status Report 2017* [64]. The report reviewed domains and indicators in the New Zealand government’s Positive Ageing Strategy [65,66] and the WHO’s AFCC [16] and provided useful baseline evidence to cite in support of on-going age-friendly advocacy.

The eight seniors (three Māori and five European/Pākeha) selected to serve on the second SAP (2017–2019) were united in their quest to progress the age-friendly agenda, and on 10 July 2018 the governing body’s Environment and Community Committee resolved unanimously to join the WHO’s global AFCC network [35]. Subsequent resource allocation by the council enabled Auckland’s AFC Project Community Engagement work to begin. Connected to the Belonging and Participation strategic outcomes in the Auckland Plan 2050 [46], the AFC Project’s purpose was “to develop a region-wide cross sector action plan” [39] (p. 5). Engagement focused on eight policy domains comprising the WHO’s Age-friendly framework, with an additional (ninth) Culture and Diversity domain. Written feedback from twenty community workshops and facilitated conversations attended by over 600 mainly older residents and staff from community organisations providing services for older adults was merged with responses to two different surveys.

Survey respondents aged 15–100 years included: 449 “Have Your Say” online and translated hardcopy survey (including Mandarin, Samoan and Tongan) respondents aged 55 years and older, but mostly in the 65–84 age group, with 19% Asian, 16% Pasifika and 3% Māori respondents; 2232 “People’s Panel” online respondents, mostly European/Pākehā female, with 5% Māori, 4% Asian and 3% Pasifika respondents. The 21% People’s Panel response rate was considerably higher than the usual 12% survey response rate and demonstrated the significance of the AFC Project for Aucklanders. Community feedback revealing the needs and aspirations of older residents is summarized in the Findings Report [39] and presented in Table 3 below.

The merged community feedback was condensed into four conceptual pathways that offer exploratory opportunities of working together for lasting change [67]. The four pathways included: (1) Enabling greater connection between older people and the world; (2) Recognising the individuality and diversity of older people; (3) Creating clear and accessible information pathways; and (4) Making everyday life easier for older people. Since the establishment of the inaugural SAP in 2014 Panel members have traversed each of these pathways in their advocacy and advice proffered to create and enhance awareness of the potential intergenerational and cross-cultural benefits throughout the life course associated with age-friendly communities [4,19].

### 2.3. Framing Autoethnographic Lived Experience

As the authors of this article traversed the boundaries between researchers and research participants, it is instructive to provide clarifying details about their social locations [68]. Firstly, this article’s collaborative co-production processes involved discussing the ethical implications of participant involvement [24] and secured the informed consent of fellow two-term members of the Auckland Council SAP. Secondly, given the dearth of older co-researcher-participants in Age-friendly publications and the need to confront challenges associated with democratizing knowledge production [69], this bricolage inquiry weaves auto-ethnographic threads into the text [36]. Thirdly, the sudden and unexpected introduction of a maximum two-term limit at the start of 2020 for those serving on the council’s six Demographic Advisory Panels raised concerns about the lack of continuity in community leadership on the SAP at a time when developing the AFC Action Plan would involve “Working with council teams, community partners and organisations, informed by the community engagement to develop a plan that will make a real difference to older Aucklanders” [39] (p. 9).

### 2.4. Interview Method

The dynamic flux of past, present and future collaborative interactions adds a temporal structure to this inquiry [30]. Temporal flux is apparent in the fifteen relational leadership interview prompts that were originally developed to glean how service-learning partnerships between community agencies and institutions of higher learning in New England (USA) evolved over time [37]. Enhancing understanding of community partnership processes and outcomes in organisations hosting tertiary students, the interview prompts focused specifically on revealing the voices of community partners to elucidate “their knowledge, attitudes and skills” so that “deeper insights can be mined about growing and sustaining partnerships” [38] (p. 2). Furthermore, three specific leadership proficiencies of: knowing (especially sharing a common purpose to facilitate empowering participatory citizenship through ethical decision making that promotes and supports community processes); being (especially expressing hopeful commitments to socially responsible power sharing that promotes equity, values integrity and develops systems thinking perspectives); and doing (especially involving others in co-creating visions to identify goals, build coalitions and nurture reflective learning capacities) proved to be foundational in collective cooperative relationships among people striving to achieve positive change [70].

The semi-structured interview guide development followed Kallio et al.’s (2016) recommended phases [71]. Doraldo and Giles’ (2004) fifteen prompts were identified as suitable for the current research setting and adapted by the lead author who changed the generic reference in the prompts from ”the project” to “Auckland Council’s Age-friendly City (AFC) project” (refer Table A1 in Appendix A). The lead author then used these adapted prompts in semi-structured individual interviews of three fellow two-term SAP members, to elicit their in-depth responses about Auckland Council’s AFC Project. The initial interview of the SAP (2017–2019) chairperson as a key informant affirmed the internal validity of the interview protocol. The subsequent two interviews demonstrated that the protocol was flexible enough to allow for clear differences and similarities to emerge and for the interviewer, who was very familiar with the topic, to probe further, where appropriate [71].

### 2.5. Participants

**Figure 1:**
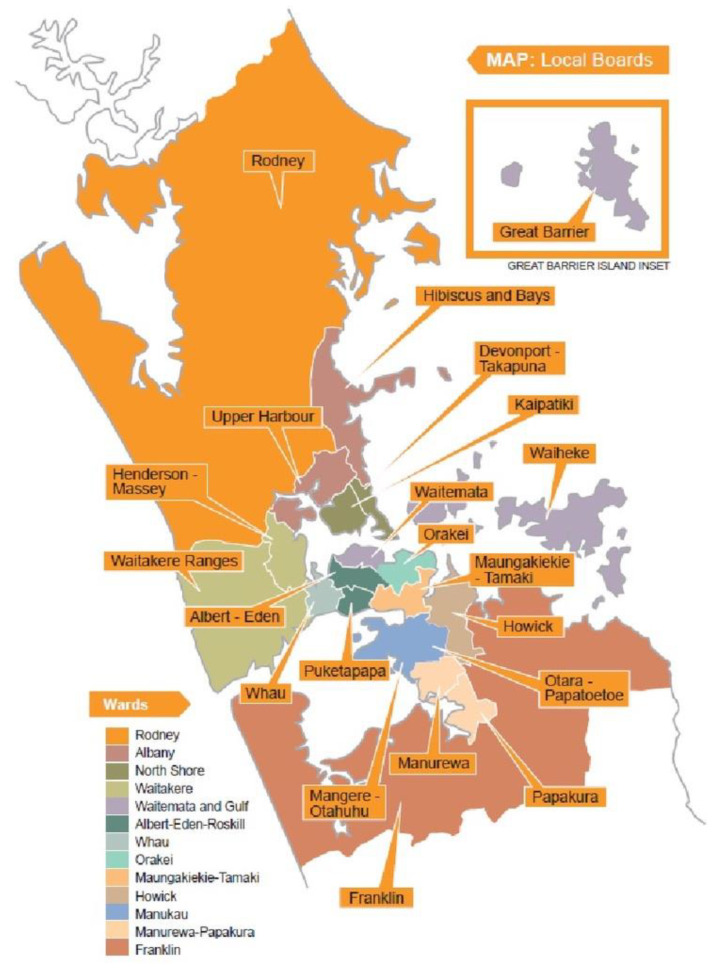
Map showing the locations of Tāmaki Makaurau’s 21 local boards. Map source [72].

Participatory inquiry processes involved discussions amongst four older Pākehā (European) community leaders, who served two terms (2014–2016 and 2017–2019) on Auckland Council’s Seniors Advisory Panel (SAP) over a period of six years. Aged between 71 and 93 years, two of the three female research participants (the oldest, J.L.R., and the youngest, lead author, J.B.) reside, respectively, in the Kaipatiki and Hibiscus and Bays Local Board areas on the North Shore; the third female (co-author J.C. who is 87 years) lives in the Waitakere Ranges Local Board area to the west of the central city and the male research participant (R.F.—aged 72 years) lives in the Māngere-Otahuhu Local Board area in the south (See Figure 1 below). J.C. and R.F. are New Zealand born, whilst J.L.R. and J.B. are South African born migrants who settled in Aotearoa New Zealand 30–40 years ago.

Face-to-face interviews were conducted in late February 2020 in J.L.R. and J.C.’s homes, and a telephone interview with the third community leader (R.F.) in early March 2020. The three audio-recorded interviews ranged in length from 33:58 to 48:22 minutes and were transcribed verbatim. Each participant received their verbatim transcript to verify and edit, prior to confirming their informed consent that the lead author apply a relational leadership model (RLM) lens [38] to extract evidence of leadership components and narrative themes from their transcripts.

### 2.6. Data Analysis

The visible expressions of older community leaders’ ways of knowing, being and doing in the current inquiry context [38] are linked to the five core leadership components identified by Komives, Lucas and McMahon (2013) in their relational leadership model (RLM) [70].

A narrative analysis of the verbatim interview transcripts afforded the co-authors opportunities to explore how individual and collective community leadership experiences influenced and supported advancing the SAP’s shared age-friendly vision for Tāmaki Makaurau Auckland [73].

The transcripts were initially read and explored individually to glean an understanding of their emergent structure and thematic content. That procedure offered valuable insights about collective collaborative processes and networks which established pragmatic strategic links [74] to set aside roadblocks and facilitate the activation of age-friendly initiatives within the council and across the region.

The three transcripts were then merged to create a combined (collective) transcript structured in question order, which was shared with the co-author. The lead author read through a printout of the combined transcripts and coded the text to identify the prevalence of five different RLM leadership components (purposeful, inclusive, empowering, ethical and process-oriented) in the transcribed responses. A framework analysis grid [75] comprising 15 columns (one for each interview prompt) and five rows (one for each RLM leadership component) was populated column by column with the initials of those participants whose responses revealed one or more of the five RLM leadership components. The resulting visual synopsis revealed the leadership component response profiles of each participant and the collective distribution of leadership components within and across the 15 interview prompts. A visual inspection of the patterns of collective leadership components across the 15 interview prompts offered evidence of the influence of dynamic temporal flux across the participant responses, which were grounded in the present but reflected on the past and anticipated a more age-friendly future [30].

## 3. Results

Despite sharing a common vision of an age-friendly Tāmaki Makaurau Auckland, noticeable differences emerged in the purposeful intent and focus of the three participants’ interview responses; an observation that provides glimpses of the nuanced nature of diversity in later life [76,77]. Accordingly, the results initially focus on the differing community leadership experiences and divergent expressions of knowing, being and co-constructing age-friendly processes and initiatives [38]. The collective narrative offers shared insights about temporal flux and loss in momentum of the AFC Project, in addition to mitigating resistance to progressing an AFC agenda, and the generative value of lifelong learning [78]. Table 4 (below) summarises these findings.

### 3.1. Heterogeneity in the Age-Friendly Relational Leadership Interview Narratives

Participant narratives are presented in the chronological order the interviews were conducted. The narratives begin with the first two interview prompt responses (Q1 and Q2) that describe each participant’s role/personal involvement and their motivation for participating in Auckland Council’s AFC Project. The subsequent narrative structure reflects insights gleaned from participant descriptions of and reflections on their prior experiences in community projects similar to the AFC Project that mainly focus on their Q10 and Q11 responses. Details about relevant age-friendly contributions of community partners and networks (Q3 and Q12 responses) conclude and provide a backdrop for the subsequent focus on the dynamic temporal flux noted, especially when encountering and mitigating resistance to integrating AFC concepts within the council.

#### 3.1.1. Constructing Purposeful and Ethical Relational Leadership

The Chair of the 2017–2019 SAP was the first participant interviewed. J.C.’s introductory responses revealed her purposeful, yet patient intent evident from the outset in her role as chair of the second SAP: *We both got involved at the very beginning, really, on the first panel* (which was advocating registration with the WHO’s global network of Age-friendly Cities, but encountered political and administrative resistance) … *and because of the way the (inaugural) panel was treated and the end point that first panel came to, we were so cross that we became absolutely determined to take that as our main plank forward; um, supposing they allowed us to remain on the panel*. This comment revealed J.C.’s appreciation of the fact that council processes and staff determined the SAP membership.

Referring to her prior experiences J.C. explained that she drew on a deep well of community involvement: In my 55 years or whatever it is of working in the community as an elected representative, it is a case again of conviction. I guess you have to say something about integrity, and by that, I mean having proved yourself to be able to walk the talk, as it were; come up with what you promised; never over promise. Well I had to fight for five years to make sure we got our new library … We did get our new library in the end, but we also got a set of traffic lights … there as a mark, as far as I see it, to the occasional bad things that happen in council because that was delayed; but, on the other side, by delaying it we actually got a better outcome all around. Ethical and process-oriented leadership components evident in the interview extract above typify J.C.’s open and transparent collaborative interactions to facilitate achieving empowering community outcomes.

Reflecting on any potential lessons learned from past experiences J.C.’s empathetic relational intelligence [79] and awareness of connecting with community members was readily apparent: *I think I just worked in my normal way of working, which is collaboratively. I’ve always tried to be empathetic where necessary. And I’ve had that comment made to me too, before, about the way I’ve worked with people. Because when you’re chairing things like the annual plan or the long-term plan in committee and you see people coming in on a regular basis, you know what’s going on sometimes in their lives. And that’s where you can show that empathy without it looking like you’re trying to be smart. Because you’re not. You’re only trying to help them cope with what’s going on.… And it’s community. And community is so important. It doesn’t matter what size it is. Start at the bottom; small and grow. That’s where you go.*

J.C.’s deep knowledge of central and local government ecology and networks salient for progressing an age-friendly agenda was instrumental in developing and strengthening alliances and processes to mitigate the resistance the inaugural SAP experienced. She explained: *Well we were fortunate because the government had moved, with Hon. Tracey Martin (Minister for Seniors), and had already got themselves into the AFCC network on behalf of the country, which in theory should make it easier for others to follow. I’m not sure that’s necessarily the case, but still it’s a good point because we knew then that we had support from a ministerial level.*

J.C. went on to describe collaborative process-oriented doing actions the SAP took to ensure that the politicians and council staff were better informed about the WHO’s AFCC network, and the staff appropriately resourced to deliver what was required: *The Mayor and council we felt had to be convinced, and so we set about putting in place a strategy to convince them. Staff were a different matter, because of course, their masters are the council. And we had no obvious way to change their role, because it’s quite prescriptive—their work programme is set and funded. We were no longer in quite the right way included within their work programme. What we wanted to happen was not happening in the way we would like it to. As for the external partners, it depends I suppose who you’re looking at? Service providers or “other stakeholders” in inverted commas, some of which are not actually service providers in the true sense, but I suppose they are providing a social service to their members. Grey Power is just a club, really, isn’t it, with a special focus? But Age Concern is supposedly supplying services to the cohort that it should do.* Grey Power is a voluntary national organization founded in 1986 to support seniors’ welfare and wellbeing [80]. Established in 1948, Age Concern New Zealand promotes wellbeing, rights, respect and dignity for older people [81]. J.C.’s comments about “other stakeholders” revealed her awareness of the need to be inclusive of a range of stakeholders within the sector.

Reflecting on the length of time J.C. had been in local government and the diversity of her extensive social network she observed: *Yes, my daughter-in-law used to say: ”Somebody knows you wherever you go”. But it’s not really quite the same now, because I’ve moved sort of back a bit; but it does help. That sort of thing does help … because you can join the dots.* When the interviewer (J.B.) cited the importance of crystal-clear communication to avoid projects failing J.C.’s response revealed a purposeful “can do” leadership component: *Exactly. Well, I’ve seen that happen too in other organisations, and wouldn’t like that to happen to anything that I was involved with, because you don’t put your energy into something for it to die.*

#### 3.1.2. Voicing Hopeful Optimism for More Inclusive Local Age-Friendly Activities

The oldest participant’s responses drew on over half a century of social work and community development roles setting up local projects; activities J.L.R. has previously described as similar to setting up Chinese spinning plates, as they require checking every so often to ensure they’re still spinning [82]. Responding to the initial interview prompts J.L.R. remembered: *I was very much involved in thinking of what an Age-friendly City was.* She explained that well over a decade ago, when the AFC advocate Dr Alex Kalache visited Aotearoa NZ: *There was a group of men who were involved in the council and we put it (the AFC concept) to them. And they discussed it. It was just discussion at that time. But it was ”What could we do to promote Age-friendly?”. Meaning there would be activities which would be for older people and we felt not only for older people, but for everybody—that it would serve the community. And that I remember was the main thing.* In the interview J.B. responded, noting J.L.R.’s inclusive intergenerational approach was evident right from the start, and a characteristic feature of her feedback to council. J.L.R. explained that she had joined the SAP: *Because I have always been a member of the subsidiaries (that influence the council), and I knew my involvement was to represent the community.*

When asked to identify any factors that might contribute to the success and/or failure of the AFC Project (Q9) J.L.R. made the first of four references to the North Shore’s Older Women’s Network (OWN), which was established in 1990 by a group of older women and ”seeks to enrich women’s lives” [83]: *It’s alright to say Auckland is an AFC but they’ve done nothing that says ”Oooh look we’ve organised this group and we’ve organised that”. We’ve got this Older Women’s Network or OWN. OWN members went to the next city (Hamilton); we caught a boat and we did this, but we organised everything; whereas I think the council could have.* OWN featured again when J.L.R. described her prior experiences that were similar to the council’s AFC Project (Q10): *Um, through OWN we have organised events, trips,* etc. *But nothing to do with Auckland Council.* Responding to the prompt about AFC Project partnerships (Q12), OWN again became the focal point: *I would take it as the Older Women’s Network, OWN, that have kept meeting to do the things that we spoke about in those days. And in fact get it done.* J.L.R.’s comments revealed her expectations that an AFC should include council staff and processes supporting age-friendly community groups such as OWN to arrange relevant activities for older residents. In response to the Q11 prompt asking if any prior community project experiences influenced her current behaviour J.L.R stated: *I still don’t think that Auckland is an AFC, but I haven’t given up hope* ... *that’s why I think, like having the Older Women’s Network that are doing things and still having events, does give it (the AFC concept) a link.*

J.L.R.’s hopeful optimism about Tāmaki Makaurau Auckland becoming an AFC—*There’s no reason not to*—was expressed in a number of her interview responses: *It’s time to remember the very first time I heard (about) the AFC I felt invigorated. And um, I thought “Let’s do THAT!” And the others weren’t so enthusiastic*. (Response to Q5 prompt discussing initiating and/or securing commitment from partners to advance the AFC Project goals.)

#### 3.1.3. Empowering More Inclusive Access to Transport Mobility

A retired director of the Māngere-East Community Learning Centre, R.F. explained his motivation to participate in Tāmaki Makaurau Auckland’s AFC Project: *As one reaches the ripe old age into the 60s and 70s you start to realise that often senior citizens are nearly non-citizens; or nearly invisible, or not taken account of. And it’s that stark reality that this isn’t good enough; that our city and our society should be all inclusive and involve people from diverse backgrounds and ages.* R.F.’s introductory statement revealed his awareness of the need to challenge unconscious ageist assumptions [84,85].

When describing his involvement on the SAP, R.F.’s central focus on transport mobility and accessibility issues highlighted the significance of inclusive and process-oriented relational leadership components in his responses: *I was engaged with Auckland Transport’s (AT’s) Passenger Transport Accessibility Group (PTAG)—a disability group pushing for better and easier and safer access, mainly for public transport around the city.* Responding to the interview prompts about prior community leadership experiences (Q10 and Q11) R.F. reported: *Probably my experiences … with the community opposition to the proposed east-west motorway. To me that showed how well that people can get together and have a major impact on a major bad decision, if you like, from the government and AT. And I’ve been involved in many campaigns over the years.* R.F. described a recent campaign which re-established a Post Office and Kiwibank in Māngere’s local shopping centre, only to discover the landlord (a supermarket chain) wanted to evict the two service organisations to establish a $2 shop. R.F.’s community activism focused mainly on being empowering and ensuring strategic “doing” actions achieved the identified goal. *We organised a big public protest. We had about 200 people rallying outside the shopping centre, and parked two of our community centre buses in the car park and announced that if the supermarket didn’t rescind the eviction notice on our Post Office we’d be running a free bus service, an half hour bus service for their customers, to take them to a rival supermarket on the other side of Māngere to do their shopping and within 20 min I got a call from the supermarket to say they’d drop their eviction notice from our Post Office. So these examples of people power and campaigning gave me heart that when push comes to shove people can get together and have a major impact on, again, poor decision making, to put it mildly.* R.F.’s account included evidence of all five relational leadership components and revealed how an older community leader who exercised his citizen role in search of shared intergenerational objectives secured improved environmental and community outcomes [3].

Responding to the interview prompts about community partners, R.F. reported that he mostly communicated with those involved in PTAG and AT staff. Aware of R.F.’s passion for promoting the benefits of free public transport (PT), J.B. also inquired about that topic; R.F. responded: *Yes, well I wrote a paper around that, highlighting the Seniors’ Super Gold Card* [86] *and how it had changed the lives of so many senior citizens being able to easily get around the city and do what they want to do; whereas previously that was a major hurdle for a lot of people, just getting out and about, socializing and engaging. So if free public transport was so successful, I argued, for senior citizens, why should it not be expanded throughout the rest of society? And perhaps starting with school students and tertiary students and slowly expanding it? I realise that free PT is not something that could be implemented over night; it will involve a lot of planning and probably be more successful if it was implemented stage by stage, and hence the suggestion of starting with students as the next stage after the successful gold card experiment with seniors. We discussed that on the SAP and that concept got warm endorsement and I took it further to AT, citing examples from overseas where free PT has been expanded to citizens successfully in other cities. And as we speak it’s expanding now quite rapidly around the world, where more and more municipalities are introducing or considering introducing free PT across the board, to really get people mobile; not just senior citizens, but everybody—with a view of not just mobility and accessibility but also to seriously cut pollution, traffic congestion and as a way of combatting climate change.* The intergenerational focus in R.F.’s response demonstrated an inclusive leadership component, whilst the reference to combatting climate change provided process-oriented systems thinking perspectives. At the beginning of the 2017-2019 SAP the members identified ”protecting our environment for future generations” as one of five priorities in their work programme [35].

R.F. concluded his responses about his prior experiences as a community leader with a statement discussing the benefits that accrue for seniors accessing free PT that revealed ethical and inclusive leadership components and expressed collectivist values: *It’s not good enough to say ”Oh well I’m all right Jack. This is great. I can get around”. Instead of taking that attitude and saying ”This is so good, and so beneficial; obviously beneficial, why should it not be spread across the board?”.*

### 3.2. Dynamic Temporal Flux and Loss in Momentum

Research understanding older Aucklanders’ attachment to their social spaces recommended that future inquiries explore their experiences of connections across time [87]. Temporal dimensions such as changes in rhythms and pace as people age [30] can be overlooked. For example, this response embedded in J.C.’s interview narrative reported above: *But it’s not really quite the same now, because I’ve moved sort of back a bit* provides a glimpse of how an individual’s reference to a change in pace can pass undetected if the text is scrutinized through a lens that ignores changes over time.

All three participants interviewed explicitly expressed their concerns about the loss in momentum of the AFC Project. For example, responding to the prompt that probed awareness of any personal or organizational risks associated with the AFC Project (Q7) J.C. commented: *The only risk now is that the momentum is kept up because we are not there yet by a long shot. We are only still at the beginning of working out the work programme that’s got to go by the end of this year (2020) to the World Health Organization. And it worries me a little bit because it could have been done more quickly to have got the basic work programme there, and then expanded as you went along; each annual plan you could add to that, and so forth. But it didn’t seem to be picked up that way. I think that it needs freeing up a little bit. It’s a bit tightly held and I think the more important thing to make sure that happens is that they choose a governance set up which is not just service providers. You’ve got to have someone who is going to monitor what the service providers provide, and they can’t monitor themselves. It cannot be just the SAP, because that has no full credence or life span. It will need to be a group of people, not randomly selected, but people with some knowledge of the life and times of people over sixty-five.* J.C.’s assessment of risks associated with the council’s current top-down approach aligns with Torku et al.’s (2020) observation that previous top-down implementations have failed to support AFCC initiatives, whereas closer collaboration between partners at the flax roots and local levels have been effective, particularly in resource-scarce cities and communities [20]. J.C.’s insightful stock take included a strategically mindful consideration of the council being willing to share power with seniors and explore sustainable future governance options [88,89].

R.F. also raised his concerns when responding to the prompt asking about the AFC project’s successes and possible failures: (Q13) *Yeah, I’m very disappointed with the decision to not allow members who have been involved for two terms because that has the effect of cutting the continuity ... To me this is like a movement: things grow, and to cut them short, you know, unnecessarily and artificially, has the risk of stopping that continuity; stopping the momentum if you like for age-friendly prospects in the city. And yeah, I see that as a major risk and I’d suggest that perhaps one way around that if council can’t be persuaded to change that policy, which was again announced without any discussion (with panel members) that I’m aware of, that there should be consideration to setting up a vehicle to continue that momentum from the previous panels, and perhaps that could be in the form of setting up some sort of lobby group which would no doubt be independent from the council because it would no longer be a council body so to speak ... Yes that’s a good start, but my worry is that it’s just going to dissipate and fall away, again because of the likelihood that there’s little or no momentum to carry it forward. These things don’t happen just out of the blue. They happen because people want them to happen, and so there’s got to be that ability to continue pushing for these ideals of an AFC and everything that goes with it, otherwise it’s just going to end up as a lot of nice fuzzy words and no action.* Golant’s (2014) critique of age-friendly communities warns of the dangers where the prime catalyst for initiatives is strong leadership and not community need [89]. R.F.’s involvement in his local community centre, and his activism within that community suggests he would be an ideal actor to participate in the development and implementation of age-friendly initiatives in South Auckland [90].

J.L.R. responded to the Q13 prompt asking how successful she felt the AFC Project was to date? So far as I know Auckland Council are not involved in age-friendly. Maybe they are and they contact younger people, but I don’t know. The rationale that informed J.L.R.’s comment emerged when she responded to the next prompt asking that she indicate ways in which the AFC Project has or might yet fail to achieve its goals: I don’t know who on Auckland Council of their staff is responsible (for the AFC Project), because if they were we would know and could try and contact them; but we don’t know if there is anybody. We certainly haven’t been told what they’re considering. In fact I know very little of council, because they do not correspond with ”outside”. You know, there’s just Auckland Council and that’s all you know; but they don’t contact groups. J.L.R. continued that response thread with a future focused suggestion about the opportunity for council staff to sustain reciprocal relationships with previous panel members: Especially if some of us have been involved, I would have thought that if you get people who have been involved, just for a meeting to say what could you suggest for the future? That would be something. And we’d feel that they’d take advantage of what we learnt and so on and so forth. J.L.R.’s reflective insights about paths of engagement in partnerships resonate with Doraldo and Giles’ (2004) conclusions that partnerships evolve over time and that institutional factors mediate how committed partnerships are to their relationships and the degree to which actions and interactions reveal learning (e.g., finding out, discovering, understanding), aligning (e.g., reviewing, reconsidering, re-assessing) and nurturing (e.g., cultivating, cherishing, encouraging) behaviours [37].

### 3.3. Mitigating Resistance

The Q4 interview prompt asked the participants to describe any resistance they had encountered to the council’s AFC Project, and what methods they used as AFC champions to overcome them? J.L.R.’s response focused on the reactions of the initial group of older men who had first been introduced to the AFC concept by Dr Alex Kalache over a decade ago: *I can’t say they supported it. But they didn’t, uh they didn’t talk against it.* However, a few moments later she added: *I think there was (resistance), but I knew I was right. You know I’m very short, so people aren’t scared of me, because I’m down here, and …* Joan chuckled sharing this observation. J.B. asked “How do you overcome that? How do you grow in stature? Do you match them with a sharp mind?”, J.L.R. responded: *No. I, I uh ask for their help.* J.L.R. ‘s community development experiences revealed her knowledge about ways of being and doing including forming alliances and building community partnerships to navigate operational pathways through entrenched power structures.

R.F.’s Q4 interview prompt was framed around accessible mobility in public transport (PT). His response explained how a few years ago Auckland Transport (AT) invited residents to send in contributions to what they called “The BIG Idea”. *So I took that opportunity, after discussing it with the SAP and also with the disability panel, of taking my paper proposing free PT for all in Auckland … and sent it to this quest for a BIG Idea at AT. And it got dismissed as being far-fetched.* R.F. did not mention any specific tactics to overcome the resistance he encountered at the time, but later noted how AT had subsequently changed their position and were now beginning to offer opportunities to access free PT (see for example AT’s free child weekend fares action [91]). The Ministry of Transport’s 2018 strategic outcomes framework [92] “to improve people’s wellbeing and the liveability of places” (p. 3) currently facilitates implementing initiatives such as the “far-fetched” action that R.F. had proposed.

J.C. responded to the Q4 prompt by immediately introducing a pragmatic process-oriented way that the SAP had mitigated resistance in 2018: *Well the whole (AFC) Project—when we picked it up in that second term—was to bring the council around to seeing that they had made a wrong move in sticking just to do their internal work, and not become more linked to the world-wide problem of ageing citizens. So we set about proving that to them by showing there was great support; or we felt, greater support than they did, for AFCs to be embedded in council’s work programmes on every single level and we’ve still got a really long way to go there. But that’s what really drove us.* Asked to clarify whether her reference to council meant the elected representatives or the council staff, J.C. continued: *Both. I’m talking about both, because one leads the work of the other, so therefore we had to go to council to convince them to move in a certain direction, which was a little more structured than they had been. And then for the staff to pick that up; do the work, not only for the actual programme that we want to put in place which is to join the network, but also make sure that the whole staff of council and all its many off shoots, the CCOs (Council Controlled Organisations) and so on, have the same methods and that it is embedded in all their thinking that they must cater for the tsunami of aged people that are now going to sweep the world. And I don’t think they realise what’s coming at them. And young ones, not disparaging young people at all, they just don’t understand because they’re not there yet. And they probably have grandparents that have not yet reached there either.* J.C.’s response highighted the value of considerable lived experience in local government settings and reflective understanding of age-appropriate strategies required to assist older people navigate ways of being and doing in urban environments.

### 3.4. Lifelong Learning

Responding to the Q9 interview prompt about identifying any factors contributing to the success and/or failure of the AFC Project, J.C. observed: *I think that the failure last time (when we did, at the end of the first panel’s term), when it was, well let’s say it was side tracked, I think part of that was that there was not sufficient interaction with the political side of council to explain what the differences were (with an AFC); which brings us to why we (the SAP) had the forum (in March 2018)* [93]. J.B. noted that the 2018 “Focus on the Future” Forum seemed to be a great education tool for the elected representatives and multiple stakeholders who attended, to which J.C. responded: *Well it was good; it was good, and we did have some political buy in to that too. I think it was just unfortunate timing—right at the end of the council’s term that it (the rejection) happened. Um and it goes to show that you really do need, although you’re not there to be political in the sense of the council’s workings, you do have to have engagement with political people so that they do understand what you’re trying to achieve. ... We had a new mayor, one who had agreed that he thought becoming an AFC was a good idea, because he came to the forum and learnt. We were able to present that to him and to the chair of the committee with whom I’d worked for many years, and that certainly did no harm either. So it does help to have people who I guess know the ropes a little bit better, and can, without being over bearing, can work with people; because you get nowhere unless you have good team work. And I think that the SAP had pretty good team work, on the whole.*

The Q9 response extract above demonstrates the value of the lead author being embedded in the inquiry context and reflecting on J.C.’s knowledge of networking processes gleaned from “within” [30], which enabled beneficial partnership alignments to optimise her relational leadership influence and thereby gather political support for the AFC concept [73]. In his interview R.F. observed that under J.C.’s leadership the SAP had enjoyed *healthy democratic discussions ... that J.C. encouraged that open debate and discussion instead of stifling it (because) she could see the (AFC) vision and encouraged others to share that. J.C. also sees the bigger picture. Instead of having a narrow view, we’ve tended to adopt a broad and futuristic view.* R.F. also mentioned that he had attended two international conferences in Tallin, the capital city of Estonia and major flag bearer of free PT, to learn more about the topic.

The final three interview prompts elicited “Reflecting and learning” process-oriented leadership components. Responding to Q13 about the success of the AFC Project to date (i.e., the beginning of March 2020), J.C. observed: *Well, as I say, we’ve been successful up ‘til now and now we’ve just got to make sure that people understand how to take it forward. I think that education is the key. Not just to—I mean old people themselves need educating, in some aspects, don’t they? Lifelong learning—there’s no question about that. None of us know it all, and we should be in a position where we can share and enhance the lives of our fellow travellers as much as we can.* Peter Kearns’ (2018) focus on late life learning draws attention to the increasing importance of intergenerational learning together to nurture our “living and growing humanistic heritage” [94] (p. 44).

## 4. Discussion

Understanding the complex and dynamic relationships between the global phenomena of population ageing and urbanisation has captured the attention of public policy analysts [95] and spawned an impressive and expanding body of age-friendly research activities [22,96,97,98]. Influenced by activist scholarship and research within Aotearoa New Zealand that gathers and provides evidence to advance social justice and equity agendas [27,99], the inquiry’s applied bricolage methodology has pieced together [100] contextual information [19] with lived experience perspectives of Tāmakai Makaurau Auckland’s emergent and expansive age-friendly terrain [25].

Co-constructing Tāmaki Makaurau Auckland’s evolving AFC Project narrative with fellow AFC champions has required careful attention be paid to meaning-making, as the participant-researchers were/are embedded in the inquiry setting. On the other hand, the co-authors have explored open collaboration as we/they coincidentally pursued later life learning activities. Adopting an interpretive bricolage approach within an activist scholarship paradigm [99] required that all four participant researchers understand the interactive nature of the inquiry processes [25], and that the co-authors in particular examine how their personal histories and commitment to advancing social justice agendas that support active ageing shaped their inquiry interactions [73]. Lived experience in their respective fields of local government, education and research informed reflexive scrutiny of their positioning within the current inquiry setting. Their reflections revealed a shared aim to co-produce knowledge that reported on the evolving AFC Project to date (until March 2020) so the text could be used to inform, support and, where appropriate, challenge on-going AFC Project processes [99].

Applying an equity lens to the layered bricolage pieces that describe Tāmaki Makaurau’s whakapapa resulted in the lead author intentionally privileging the significant roles that indigenous Māori and Pacific Peoples play in the city [27,100]. Although 5.0% of Māori and 5.6% of Pacific Peoples were aged 65 plus in 2018, those numbers had increased considerably since the 2013 Census (by 45.9% and 38.7%, respectively). Cognizant of the future implications of these structural demographic features, Salesa recommended the audience at his 2018 Michael King Memorial lecture embrace Pasifika values including ”speaking the language of others” [61]. The expression accentuates plurality by acknowledging the linguistically, culturally and geographically different island nations in the South Pacific [101]. However, an evaluation of three New Zealand AFCC case study sites highlighted the limited inclusion of engaging with Māori and migrant groups to develop age-friendly initiatives [102]. The Pasifika engagement process referred to as “Yavu” that acknowledges the importance of respect for Māori (Tangata Whenua) as indigenous to Aotearoa and Te Tiriti o Waitangi as the foundation for Pacific Peoples’ relationship with Tangata Whenua, offers ways to engage with diverse ethnic communities in Tāmaki Makaurau [101]. Grounded in core Pacific values of family, collectivism, respect, spirituality and reciprocity in the engagement process, Yavu offers opportunities to co-create age-friendly arenas of convergence “where social identity, environmental cosmos, and the ancestral world meet and engage” [101] (p. 6). In the current inquiry 93-year-old J.L.R.’s interview comments revealed a lapse in communications with the council. She was unaware of what was happening with the AFC Project, because the council *do not correspond with ”outside”. You know there’s Auckland Council, and that’s all you know, but they don’t contact groups.* J.L.R. had referred to the absence of any reciprocal engagement with the council earlier in her interview, when she wondered: … *maybe they contact younger people*? The reference to “younger people” reflects J.L.R.’s awareness of her communication preferences (for hard copy text, phone calls or face-to-face meetings), and the invisibility of gauging the communication needs of residents in their 90s receiving information from and interacting with council. Given J.L.R.’s strong and active involvement with the Older Women’s Network (OWN) activities, she was also aware that council had not contacted OWN to disseminate any updates about the AFC Project. Developing collaborative community partnerships and networks that enable manākitanga (welcoming reciprocity) to affirm and empower older residents and their families [103] should be a key priority when co-producing [104] a sustainable AFC Action Plan [88] for Tāmaki Makaurau Auckland.

To introduce the unique features of the inquiry’s Polynesian setting the lead author observed Māori tikanga (processes) by exploring, selecting and “placing in layers” narrative text that revealed Tāmakai Makaurau’s whakapapa (origins) [105]. Traversing space and time, the city’s whakapapa revealed relationships with both the land and the people, and acknowledged the mana (prestige) held by the region’s first people; mana that was ignored by colonial settlers [47,106]. Diverse information sources described the city’s rapid urban development [31] and current local government governance processes [32]. They also revealed how contemporary statutary obligations to honour principles articulated in Te Tiriti o Waitangi [107] have been translated into values such as Atawhai (kindness, generosity), Kotahi (strength in diversity), Auaha (creativity, innovation), Pono (integrity) and Taonga tuku iho (future generations) in The Auckland Plan 2050 [46] (p. 23), to nourish residents’ wellbeing [108]. Feedback to council planners from older residents, SAP members and organisations supporting seniors not only drew attention to the importance of including demographic ageing in the final draft of the Auckland Plan 2050, but highlighted the need for greater age-friendly awareness within and across council’s organisations. Given the patronising face of ageism that has emerged during the COVID-19 pandemic [109], taking appropriate counter measures requires careful and considered attention. One possible way to respond is to involve older people in qualitative particpatory research which can be used to co-produce evidence that challenges and influences ways in which societies construct ageing [110]. Participatory research and engagement with older people requires that attention be paid to four key areas: Mahi tahi (collaborative and equitable involvement) to address and resolve issues of power imbalance; Kotahitanga (solidarity and capacity building) which may require co-learning through the exchange of knowledge and skills; Rangatiratanga (empowerment and action for social systems change) which is one of the purposes of participatory research and aims to inform and facilitate taking civic action because of increased community awareness [99] and Kaitiakitanga (sustainability) to optimise opportunities that develop supportive infrastructure for on-going participatory research [110]. Including process-related actions that improve council’s engagement and communications with diverse community networks (including Māori and ethnic communities and those aged 85 years or older), should be included in Tāmaki Makaurau’s evolving AFCC Action Plan.

Using RLM interview prompts to ”hear” older voices [111] enabled the three AFC champions to share their lived experience as community leaders with the lead author [104]. Commenting on mitigating resistance within council to age-friendly concepts J.C. also drew attention to the need to develop on-going age-friendly awareness and training programmes that should be embedded in all council’s thinking, services and processes *so they are ready to cater for the tsunami of aged people*. The *Age-Friendly Community Evaluation Report* prepared for New Zealand’s Office for Seniors [102] concluded with a similar recommendation about the educative role the Office for Seniors could play “to ensure central and local government and communities understand what age-friendly means” (p. 2). Informed by community insights gleaned from the AFC Project’s Key Community Engagement Messages (refer to Table 3) [39], the Office for Seniors and the council could partner with creative agencies to co-produce an innovative education programme that challenges invisible yet pervasive ageist assumptions within the community [109,112]. Developing an age-friendly accreditation for diverse organisations across the region (see for example the Arts Council England’s Age-Friendly Standards [113]) could likewise ensure that venues hosting popular intergenerational events such as Auckland Conversations [114], the Auckland Writers Festival [115] and cultural or sports events are welcoming, accessible and age-friendly.

In his interview R.F. highlighted that a significant downstream impact of cutting the continuity of AFC leadership on the SAP has the risk of stopping the momentum for age-friendly prospects in the city. He added that there should be some consideration to setting up a vehicle to continue that momentum from the previous panels ... *which would no doubt be independent from the council, because it would no longer be a council body*. J.C. expressed similar concerns when she observed that she thought the evolving AFCC Action Plan needed to free up a little bit: *It’s a bit tightly held and I think the more important thing to make sure that happens is that they choose a governance set up which is not just service providers*. J.L.R.’s comments that so far as she knew council are not involved in age-friendly because she had received no communication about what was happening highlights the need for council to develop a more outward focussed communications policy that is inclusive of our oldest residents. J.L.R.’s astute observation about council’s loss of AFC relational leadership knowledge on the incoming SAP illustrated that council had failed to consider the need for succession planning that enabled knowledge transfer for the incoming SAP members: *Especially if some of us have been involved, I would have thought that if you get people who have been involved, just for a meeting to say what could you suggest for the future? And ... take advantage of what we learnt* (over the past six years). It is noticeable that the evaluation of the three NZ AFCC sites identified that “A committed steering group was central to the process of implementing an age-friendly programme” and that “managing community politics was a necessary skill required by the steering group leadership” [102] (p. 30). On-going collaborations with seniors to enable the co-production of flax roots age-friendly initiatives are important [20]. However, as the interview narratives have revealed, the top priority for the council is to create an appropriate AFCC governance structure that embraces the values articulated in the Auckland Plan 2050 [46], embeds the principles of Te Tiriti o Waitangi within the structures and processes [27,108], and empowers active ageing for all across their life span.

The theme of lifelong learning [116] emerged when “listening to hear” the voices of the SAP’s AFC champions [111]. J.C. referred to the educational value of the SAP’s 2018 “Focus on the Future” Forum, which was attended by over ninety older residents and encouraged expressions of active citizenship, through an agenda that included stimulating presentations on a variety of relevant age-friendly topics [3] and World Cafe group discussions [117] to elicit and shape feedback for the Auckland Plan 2050 [46]. ”Opportunities for lifelong learning” was also a key Auckland Project community engagement message, under the AFC Project Framework’s Civic Participation and Information Domain (refer Table 3). Commenting on the success of the AFC Project when she was interviewed at the end of February, J.C. expressed concerns about how to take the project forward? She recognised that not only council staff need to become better educated about AFCC, but older people themselves: *I mean old people themselves need educating, in some aspects, don’t they? Lifelong learning—there’s no question about that. None of us know it all, and we should be in a position where we can share and enhance the lives of our fellow travellers as much as we can.*

Kearns’ vision of a learning city, where learning in later life is nurtured and community centred, recognises the possiblilties of seniors with lived experience, time and relationships developing into community leaders [94]. The vibrant growth in the network of “Third Age” U3A branches across the Auckland region since the establishment of the first branch in 1989 reveals how opportunities to extend personal learning through collaborative research, discussions and field trips provide personal and social benefits for members [118]. Kearns also predicted the increasing importance of enabling intergenerational learning to transfer shared cultural values [94], which is reflected in Auckland Libraries’ strategic plans [119] and the ninth “Culture and Diversity” Domain of the AFC Project that was added to the WHO AFCC Framework [14]. In their roles as SAP members both J.L.R. and R.F. articulated strong support and advocacy for diverse intergenerational age-friendly initiatives. A successful intergenerational transmission of cultural values from grandmother to mother to child that was established in 1982 is currently regenerating Māori language through Kōhanga Reo ”language nests”, which provide total immersion in Māori language and values for preschool children and their families. This Māori-led initiative has not only increased the numbers of people speaking te reo, but also affirmed Māori identity and empowered Māori women to engage in on-going education [120]. Opportunities exist for the emergent AFCC Action Plan to support the continuing regeneration of te reo. Similarly, the highly acclaimed Pacific Heritage artists and cultural leaders who formed the Pacifica Mamas (and Papas) collective in the late 1980s, so first generation migrants could meet to exchange stories, extend their knowledge and strengthen their Pasifica arts practice, facilitate intergenerational transmission of Pasifika cultural values through diverse community activities and performances [121]. Recent research has described the specific challenges that Pasifika families in New Zealand faced when adjusting to coping with the pandemic’s disruption, uncertainty and social distancing measures [122]. The explosion of virtual learning and demand for digital connections to access essential services such as healthcare and banking have in turn revealed that digital access and digital literacy are fundamental factors that determine older people’s capacities to sustain their agency and wellbeing during pandemic lockdowns [123]. Clearly the evolving AFCC Action Plan will need to include strategic actions to improve digital inclusion [124].

## 5. Conclusions

The current inquiry has challenged normative expectations that traditionally seniors are the objects of Active Ageing research [8]. Knowing how to transform into being active participant-researchers required seeking and learning ways to apply innovative interpretive bricolage techniques [25,26], in order to convey the multiple complexities involved in co-constructing these age-friendly Tāmaki Makarau Auckland narratives [19,110]. Guided by activist scholarship principles [27,99,106] and the co-authors’ concerns about equity and social justice, the research-participant narratives were curated to reveal past developments, capture present accomplishments and opportunities, and anticipate future priorities [19,30]. Developing co-constructed narratives of a dynamically evolving AFC Project during a global pandemic [2] has highlighted the challenges inherent in balancing the “top-down” versus “bottom-up” interactions that drive AFCC implementation processes [20].

The 2017 review commissioned to support the implementation of AFCC in Aotearoa New Zealand advised that processes and plans should have built-in flexibility, and older people should be involved in all stages of the development of AFCC processes and plans [19]. Developing a shared understanding of the implications of “being involved” in AFCC co-production processes requires greater attention be directed to clarifying where on the public participation spectrum the diverse AFC Project community engagement interactions lie [104,110]. The interview narratives of the three older community leaders and Tāmaki Makaurau Auckland AFC Project champions have provided insightful reflections on the milestones achieved as of March 2020. Their insights also offer thought-provoking case study material that could be included in service learning opportunities, to enhance ways of working together across all four conceptual pathways that were identified from the merged AFC Project community engagement feedback [67].

A number of service-learning recommendations were made when discussing the results. However, on reflection, two emerge as immediate priorities: (1) Co-develop a sustainable AFC Project Steering Group co-governance framework that embodies the values and principles of Te Tiriti o Waitangi to enable empowered active ageing for all residents across the region [27,108]; (2) Co-develop a succession plan that enables the timely transfer of knowledge and skills to empower incoming SAP members about the evolving AFC Project [125].

## Figures and Tables

**Table 3:** Age-friendly Auckland Project’s Key Community Engagement Messages ^1^.

#	WHO Age-Friendly Framework Domains
1	Outdoor Spaces and Buildings:
	▪ Accessible and safe journeys from public transport or finding parking through to getting into buildings and accessing indoor and outdoor activities. ▪ Public amenities in the places we go that are clean, accessible and well maintained. We feel more comfortable when both gender specific and unisex toilets are available.
2	Transportation:
	▪ Safe and accessible roads, footpaths, public transport, transport hubs and everything in between that accommodate different abilities and modes of transport. ▪ Our transport journey to be seamless. We need accessible parking options, seating and weather protection at stops and drivers to wait until we are seated on public transport.
3	Housing:
	▪ Affordable housing for all, across Auckland. ▪ We need housing options that are universally designed to allow us to age in place. Different housing types, models and sizes.
4	Social Participation:
	▪ We need barrier free access to transport, facilities, activities, outdoor spaces and events. ▪ Affordable activities, programmes and venues for our groups.
5	Respect and Inclusion:
	▪ Visibility, positive images, diversity and stories of older Aucklanders. ▪ Intergenerational respect and understanding—our lives, choices and diversity.
6	Civic Participation and Employment:
	▪ We need employment options and ways to transition from full-time employment to part-time work, flexible work, volunteering or retirement, which recognize our changing circumstances, abilities and the contribution we want to make. ▪ Opportunities for lifelong learning.
7	Communication and Information:
	▪ Places to access information and get affordable support and training so we can keep up with technology. ▪ Information and news about community matters, services, events and activities provided in a range of formats, across multiple channels and ideally in our own language.
8	Community Support and Health Services:
	▪ Accessible healthcare—services to be where we need them, when we need them. Mobile facilities that go to the places where we are. ▪ We need affordable healthcare services and support, including dentistry.
9	Culture and Diversity:
	▪ An open, friendly and inclusive society of all cultures, where there is care, respect and all people are valued. ▪ Opportunities for connection with our own culture, other cultures and intergenerationally.

^1^ Compiled from pages 2–4 of the Age-friendly Auckland Project Community Engagement Findings Report [39].

**Table 4:** Heterogeneity and shared insights in the relational leadership interview narratives.

Heterogeneity	Shared Insights
1. Constructing purposeful and ethical leadership (J.C.)	1. Dynamic temporal flux and loss in momentum of Tāmaki Makaurau Auckland’s AFC Project
2. Voicing hopeful optimism for more inclusive local age-friendly activities (J.L.R.)	2. Mitigating resistance to progressing the AFC agenda
3. Empowering more inclusive access to transport mobility (R.F.)	3. Lifelong learning

## Appendix A

**Table A1:** Auckland Council’s Age-friendly City (AFC) Project interview prompts ^1^.

1	Describe your role/personal involvement in Auckland Council’s AFC Project.
2	Discuss your motivation to participate in Auckland Council’s AFC Project.
3	List the partners to Auckland Council’s AFC Project and discuss the initial receptiveness and commitment of each partner.
4	Describe any resistance encountered and methods you used to overcome them as a champion of Auckland Council’s AFC Project. (Please be as specific as possible providing anecdotes or stories.)
5	Discuss the use of any strategy that helped to initiate, implement, gain commitment from partners, and in general further the goals of Auckland Council’s AFC Project.
6	Discuss any recognition you might have received for your role in Auckland Council’s AFC Project.
7	Discuss the risks (both personal and organizational) associated with Auckland Council’s AFC Project.
8	Discuss your perception of your effectiveness in facilitating the goals of achieving desirable outcomes for Auckland Council’s AFC Project.
9	Identify any factors contributing to the success and/or failure of Auckland Council’s AFC Project.
10	Describe any prior experiences you have had with any projects similar to the Auckland Council’s AFC Project.
11	Did any of your prior experience(s) influence your behaviour in this project? How? Can you please provide some examples?
12	Discuss your relationship with partners in Auckland Council’s AFC Project, and how those relationships influenced your behaviour in this project.
13	Say something about how successful you feel Auckland Council’s AFC Project is to date.
14	Indicate any ways in which you think the Auckland Council’s AFC Project has or might yet fail to achieve its goals.
15	Describe what factors have contributed to the success and/or failure of Auckland Council’s AFC Project.

^1^ Adapted from Dorado and Giles (2004) [37].
